# Research under the GDPR – a level playing field for public and private sector research?

**DOI:** 10.1186/s40504-021-00111-z

**Published:** 2021-03-01

**Authors:** Paul Quinn

**Affiliations:** Vrije Universiteit Brussel (VUB), Brussels, Belgium

## Abstract

Scientific research is indispensable inter alia in order to treat harmful diseases, address societal challenges and foster economic innovation. Such research is not the domain of a single type of organization but can be conducted by a range of different entities in both the public and private sectors. Given that the use of personal data may be indispensable for many forms of research, the data protection framework will play an important role in determining not only what types of research may occur but also which types of actors may carry it out. This article looks at the role the EU’s General Data Regulation plays in determining which types of actors can conduct research with personal data. In doing so it focuses on the various legal bases that are available and attempts to discern whether the GDPR can be said to favour research in either the public or private domains. As this article explains, the picture is nuanced, with either type of research actor enjoying advantages and disadvantages in specific contexts.

## 1. Introduction

Research is without doubt of elemental importance to the wellbeing and advancement of any society (Mirowski and Sent 2002). It contributes to scientific knowledge, economic growth and can be used to address serious societal problems. Whilst the traditional image of research is that of a university research group or university hospital, the reality is that research has always and will always be conducted by a variety of actors. A large range of private entities, varying from small organisations to large and powerful tech and social media giants are continuously engaged in research. Whilst the motives of such research may often be more commercial in nature, it is nonetheless indispensable for innovation and economic growth.

The use of data is central to all forms of research. This often includes personal data. The ability of researchers to access personal data, is often a key factor in determining whether various forms of research are able to proceed (Heffetz and Ligett 2014). Regulatory frameworks, including data protection frameworks therefore play an important role in many instances in determining not only what forms of research may be conducted, but also what types of researchers are able to carry them out (Dalle Molle Araujo Dias 2017). This article looks at the importance of the EU’s General Data Protection Regulation (GDPR) in determining what types of research can be performed with personal data. In particular it focuses on the legal bases that exist within the GDPR that can be used for such purposes. As this paper discusses, the GDPR is, in general, friendly to research and presents a number of different options in terms of legal bases (informed consent being only one) that can be used in various contexts. This article aims to demonstrate which legal bases may be of most use to various types of research actor and aims in particular to contrast the position of researchers based in public sector institutions like universities with others working in the private sector e.g. for large commercial entities.

Section 2 of this article outlines the importance of personal data and consequently data protection frameworks for research. As section 3 discusses, this importance is only likely to increase given the increasingly data hungry nature of modern research, depending on inter alia access to forms of big data (Akoka et al. 2017). Sections 4 and 5 outline the research friendly nature of the GDPR and the various options it presents for those wishing to conduct research with personal data. These legal bases may be more appropriate for use in certain contexts and by certain types of actors than others. Whilst as sections 6 and 7 discuss, certain legal bases (e.g. allowing for research that is in the ‘public interest’ or for ‘scientific research’) may be in theory open to both public and private actors alike, the de facto reality surrounding their use means that private sector entities may find it more difficult to use them. This can be compared to the possibility to process personal data for ‘legitimate interests’. This can allow research in a number of important contexts but is in general only available to private entities and can not be used by public institutions. A further important possibility is the seemingly very broad option to further process any personal data a data controller may be in possession of for purposes of scientific research so long as it had a valid legal base for the possession of the data in the first place.

As this paper discusses, it is also important to consider the de facto context of various types of research entities. Large commercial entities (e.g. tech giants, social media platforms, or eCommerce vendors) may possess enormous pools of big data they have generated in relation to their clients. This may provide a significant practical advantage in certain conexts over researchers in public institutions. University researchers may by contrast often be dependent upon agreements with external parties to obtain research data. As sections 6 &7 discuss, these variations mean that the opportunities the GDPR provides for research are nuanced and may in reality not be equally available to all types of research actors. The answer to the question of whether there is a level playing field for research actors based in public and private research contexts is therefore complex, with neither enjoying a definitive advantage.

## 2. The use of personal data is becoming more common and complex in the age of big data

The importance of data protection frameworks to research has increased greatly in recent times. This is because more and more research is conducted using personal data and also because data protection frameworks have become more complex and far reaching. These changes are linked to the increasing ability to digitize more and more personal data and to share it in a world of ever increasing interconnectivity (Meszatos and Ho 2018). In the medical field for example patient dossiers are now routinely stored as Electronic Health Records (EHRs). Such data now forms a rich source of research data for a variety of researchers interested in investigating medical issues (Jensen et al. 2012). EHRs are but one example however. A wealth of diverse sources of data have emerged that can be of use for research (Connelly et al. 2016). These can vary from social media activity, phone tracking and efforts at citizen science (where individuals collect their own data for research purposes) (Corrales et al. 2017; Quinn 2018).^1^ The ability to collect and combine any of these forms of ‘big data’ mean that researchers have a wealth of data to analyze for various research purposes. At the same time, computing power has been increasing enormously, meaning that the various analytical processes that can be applied to such data has increased greatly. This has led to an explosion of research that is solely data (or computational) based (Quinn and Quinn 2018).^2^ Such research often does not depend on physically measuring or making impositions on individuals or objects, but rather makes use of pre-existing data. The use of such large forms of secondary data may have considerable advantages, including the analytic power that such large datasets often bring and also potentially the ability to avoid problems surrounding the primary collection of data, e.g. administrational, practical, ethical etc. Whilst this world of readily available big data might facilitate research in many ways it also brings more research within the purview of data protection. This is primarily for two reasons:

(i) *More personal data that ever before is now available.*

In a digitized world individuals are able to upload and store data (on a permanent basis) to an extent that was not possible in the past. This ranges from official and important sources such as EHRs to apparently innocuous efforts at self-quantification (Swan 2013). It may include data taken from mobile phones (that can be linked to them) or relate to the information they post to social media. In other instances, it could come from a long history of online purchases. The continued creation, storage and ability to share such data means provides a rich source of material for researchers. The ability of researchers to access such data may vary depending on the type of entity they are and the context of the research in question (further discussed in section 6).

(ii) *Many forms of big data may contain personal data (even where not immediately obvious).*

In the big data world it may not always be intuitively obvious whether large data sets contain personal data within them. It may only become obvious after careful and considered inspection of the data in question. Such data may often be large, heterogeneous and unstructured. This means that it may contain personal data in ways that are not immediately obvious (Mai 2016). Discerning whether data is of a personal nature or not is of immense importance. The GDPR confirms that where data is anonymous it is not of application.^3^ This means that researchers processing truly anonymous data do not have to concern themselves with the requirements of the GDPR (Quinn 2017).^4^ By contrast however, where data in question is personal, the GDPR is of full application (no matter how pseudonymous or encrypted the data in question maybe).^5^ Given that the bar for anonymization has been seemingly set very high, it is important not to rule out that any dataset may contain personal data even where this is not intuitively obvious.^6^ This is especially true with large data sets. This is because it may be possible to combine various elements within a large data set or with data that is readily available elsewhere in order to arrive at conclusions about potentially identifiable individuals. Advances in computing power and in analytic software only increases such a possibility. Given that data is only going to become ‘bigger’, that computing power is only going to increase and that the availability of complimentary data is only going to grow, the likelihood of any large data set containing personal data is likely to increase. This factor means that it is becoming increasingly difficult to render datasets anonymous whilst allowing them to retain any useful value (e.g. for AI analysis).

## 3. The implications of data protection for research

### A. Personal data and harms

Researchers and societies in general have long been aware of the potential for physical harms to be produced from experimentation in humans (Freidenfelds and Brandt 1996; Rothstein 2010; Drabiak 2017). In recent decades, and with scientific research becoming both more complex in its use of data (including data relating to human beings) an increasing awareness has been developing of harms that can be produced from the use of information in research. This has led to the creation of various strategies and approaches that have been formulated to regulate the use of personal data inter alia in research. Some of these may apply specifically to researchers whilst others may apply to other domains from which researchers may draw their data. Confidentiality laws may for instance limit the ability of researchers to obtain personal data (e.g. from medical professionals) (Berman 2002). Other approaches such as ethical or deontological codes may attempt to restrict what researchers can do with data in terms of research (discussed further in section 6) (Tene and Polonetsky 2016). Whilst such codes may not always be considered as law, they have had and continue to have an important role in regulating the use of data in research (see section 6).

In recent decades however and particularly within Europe, data protection frameworks have come to be seen as the most important form of regulation relating to the use of personal data. They are both general and of far reaching application, applying in most contexts where personal data is used, including for the purposes of research. Starting with Directive 95/46/EC^7^ and more recently the General Data Protection Regulation (GDPR),^8^ the European Union has effectively crafted a (mostly) harmonized regime across Europe regulating the use of personal data.^9^ Data protection has arguably risen from a position of relative obscurity, to become the central regulatory framework concerning the use of personal data in research. In doing so it has arguably come to overshadow older legal regimes (e.g. related to confidentiality) that may apply. The GDPR, being the legislative initiative that is both the most harmonizing and of the most general application, is of particular importance to researchers active across numerous domains (Quinn and Quinn 2018; Peloquin et al. 2020).^10^ In terms of its general application, it does not apply to specific contexts, but rather to most instances where personal data is being processed (including but thus not limited to research). If the GDPR is of application personal data can only be processed if certain conditionality is met.^11^ Some of the most prominent forms of conditionality relate to:

(i) *Data protection principles*

The GDPR foresees a number of important principles which controllers must adhere to when processing personal data. They are of general application and usually apply whatever the legal base is for the processing of question. The principles themselves are somewhat abstract and the application to a particular context will require reflection on the part of the data controller (Mondshein and Cosimo 2019). Data minimization for example obliges data controllers to collect no more personal data than they need for the processing operation that is envisaged. The storage limitation principle requires controllers to delete data once they are no longer needed for the purposes that were originally envisaged. Other principles require data controllers to ensure that their processing operations are secure, transparent and that privacy and data protection are taken into account at all stages of processing and planning (Forgo 2017). One important principle which the GDPR allows to be applied differently in instances of scientific research is that of purpose limitation. In particular, the GDPR allows researchers more room in terms of the description of the purpose of processing they must present to the data subject. This can be used to allow a broader form of consent for the use of personal data in scientific research than may be possible for other forms of processing.^12^

(ii) *Data subject rights*

Data controllers must facilitate a number of important data subject rights, including in research contexts.^13^ These rights may, depending on the context in question, be onerous to facilitate and may entail the creation of various administrative structures. This can be costly and time consuming for researchers. Notable data subject rights include a right of ‘erasure’ (commonly known as ‘the right to be forgotten’), a right ‘to object to processing’, a right of ‘data portability’. In forms of research that require complicated forms of big data facilitation of such rights may be complex and are once again likely to require careful consideration of the context in question. One important consideration for researchers is that the legal base selected for the processing of personal data in research may determine the extent to which data subject rights apply given the ability the GDPR allows Member States to limit them in instances of scientific research (discussed further in section 4.b).

(iii) *Administrative requirements*

The GDPR foresees a number of important administrative requirements that are likely to apply to researchers, particularly when they use sensitive data. This includes the need to appoint a Data Protection Officer and potentially the need to perform a data protection impact assessment (Quinn and Quinn 2018). In addition to the requirements outlined in the GDPR, Member states may add further requirements, in particular for the processing of sensitive forms of data.^14^

(iv) *The need for a legal base*

The above requirements will apply to most forms of processing. Before processing of personal data can be contemplated however, it is necessary to ensure that there is a legal base for processing.^15^ A legal base represents a set of conditions or a context in which the processing of certain form of personal data are permitted. Without an applicable legal base researchers can not process personal data for research or any other reason. One of the most well known is that of ‘informed consent’.^16^ As sections 4 and 5 describes this forms the legal base for many forms of research. The GDPR, like its predecessor, also foresees a range legal bases that do not require consent. Some of these will permit research in circumstances where obtaining consent from data subjects is not desirable or even possible. Each of these bases will be outlined and presented further in sections 4 and 5.

Researchers conducting research with personal data must take into account all of the elements outlined in (i)-(iv) above. Whilst each represents an important factor that should not be understated, a full analysis of them is beyond the scope of this paper. The remaining focus will be on (iv), i.e. the need for a valid legal base. A considered examination of this requirement is interesting from the perspective of this paper because the various legal bases that exist within the GDPR may not be equally available to all types of research entities. Subsequent sections of this paper will analysis this in the context of modern research in order to highlight the existence and consequent importance of such differences.

## 4. The GDPR provides a wealth of legal bases for researchers

### A. Several bases are available to varying types of actor

The increasing engagement of research by data protection frameworks such as the GDPR discussed in section 3 means that the GDPR will consequently become more determinative of what forms of research are permitted and how they should be conducted. Whilst the GDPR was not created solely for the purposes of research, it was clearly a significant form of processing that was envisaged by the those responsible for creating it (Nyren et al. 2014).^17^ Importantly, it does not attempt to shoehorn all forms of scientific research into one single legal context but rather appears to recognize that scientific research, and the various motivations behind it, are extremely heterogenous. A ‘one size fits all’ notion of scientific research does not exist. Research varies in terms of its goal (‘blue skies’, ‘not for profit’, ‘commercial’ etc.), the identity of the those carrying it out (universities, hospitals, large commercial actors) and the various data subjects involved (varying from vulnerable individuals to well informed consumers) (Shmueli and Greene 2018). The regulation accordingly foresees several legal bases that can be utilized by individuals or entities wising to conduct research. This seeming assortment of options is important given the diverse nature of scientific research. As recital 159 of the GDPR states:

*“For the purposes of this Regulation, the processing of personal data for scientific research purposes should be interpreted in a broad manner including for example technological development and demonstration, fundamental research, applied research and privately funded research”*

This points to a very open mind in terms of what the drafters of the GDPR regarded as ‘research’, with a concept that does not discriminate between research of varying types (e.g. public, private or commercially motivated). Various research activities may also be permitted under grounds that are not specifically related to research. The GDPR is accordingly not lacking in terms of potential legal bases that may be available to researchers in various contexts. On the contrary, it can almost be considered to contain an ‘embarrassment of riches’ in terms of potential legal bases that can be used for research involving personal data. The most prominent are described below (legal bases or sensitive data are discussed in section 5).

#### The use of consent

Consent is perhaps the most well known legal base for justifying data processing in general. It can be used to justify the processing of personal data in an almost undefinable range of circumstances where data subjects have provided their consent. This includes a wide spectrum of research contexts in both the public and private sector. The GDPR demands that such consent must represent a clear and unambiguous indication of a data subject’s wishes and that it must be informed. It is defined in the GDPR as.^18^

*“any freely given, specific, informed and unambiguous indication of the data subject's wishes by which he or she, by a statement or by a clear affirmative action, signifies agreement to the processing of personal data relating to him or her”*

Consent therefore can not be passive and individuals must receive a minimum of information in order to be able to provide their consent. In this regard, article 13 of the GDPR specifies a number of elements that must be explained to data subjects including, the purposes of the processing in question, the identity of the data controller and how long data will be kept for (Staunton et al. 2019).^19^

Importantly the GDPR arguably foresees a slightly looser from of consent for scientific research than is available elsewhere. This is outlined by recital 33 which states that the purpose of processing can be described in less concrete terms for instances of scientific research. This may allow researchers some room where the nature of their research makes this difficult e.g. using AI or other computational processes with various forms of big data. This extra room should not be interpreted as a *carte blanche* for researchers. The purposes of research must nonetheless be described to a sufficient level.^20^ Where this is not possible consent should not be used as a legal base for processing.

Although these conditions may not seem onerous, complying with them can produce considerable difficulties for researchers. Contacting (and even identifying) all potential data subjects may for example difficult, especially where research involved very large data sets. This may entail the use of complex administrative processes and consequentially further costs (Jamrozik 2004). Even where it is possible it may introduce an element of bias, where individuals who refuse consent may represent a statistically important group (Rothstein and Shoben n.d.). For these reasons, the use of consent may not be practical in all instances where research is to be carried out.

#### Reasons of public interest

In many instances it may not be possible (or even desirable) to gain the consent of data subjects. The data subjects may be too many, too difficult to reach or the type of processing involved may be too difficult to explain. In certain contexts consent may not be ethically appropriate because of power imbalances (e.g. where the relationship between the data controller and subject makes it difficult for the latter to withhold consent) (Solove 2013; Corrigan 2003).^21^ Numerous forms of research in the public interest fall into this latter category (Donnelly and McDonagh 2019).^22^ This includes research needed to better organize public services or other forms of governance. In order to facilitate such forms processing (which are in the public interest) the GDPR foresees a particular legal base (described in article 6(e)) for processing in this context. Another important feature of this legal base that may often make it attractive to researchers is that Member States can, through legislation, limit a number of data subject rights normally incumbent upon instances of processing of personal data (discussed further below in (b)). This includes in a number of important areas relevant to research covering both a number of data protection principles (e.g. storage limitation) and data subject rights (e.g. the right to be forgotten).^23^ In addition to obviating the need to obtain consent, the use of such a legal base can be highly advantageous to researchers in a number of contexts given that it may remove serious burdens upon researchers (i.e. the need to comply with strict understandings of various data processing principles or data subject rights) (Quinn and Quinn 2018).

Whilst being seemingly wide in terms of its scope, this legal base should not be viewed as being available for all forms of research. There are important limitations. Most importantly, it is only available where there is specific (European) Union or Member State law available (Donnelly and McDonagh 2019). This usually means that legislation must exist that identifies the controller in question as being able to carry out the type of processing in question. The use of this base represents one of the areas of data protection law where a considerable margin is left to the Member States to determine the specifics. As a result, there may be considerable variation in the exact nature of this base and its availability to researchers across Europe. As section 6 discusses, the legislation available in the various Member States may vary in terms of the extent it can be used by commercial forms of research. In some Member States legislation may be phrased in a manner that seemingly limits the applicability of this legal base to commercially motivated forms of research. Where such legislation does not exist, or is not applicable to a certain research context (i.e. commercially motivated research), this legal base for processing personal data will not be available. In addition, the use of this option must be necessary (and thus also proportional) in a specific context.^24^ In other words it can not be simply opted for because of the innate advantages it may offer, but only when consent is clearly not appropriate.

#### Processing is necessary for the performance of a contract/ of a legal obligation

The GDPR recognizes forms of personal data processing are implicit to the execution of many forms of contract. Having to continually ask for consent from data subjects would be inefficient and make the goal (which both parties had contracted for difficult to achieve). Whilst one can imagine certain contexts where consumers contract with certain commercial entities to conduct research on their data, this type of relationship is not common in the public sector.^25^ Similarly the GDPR recognizes that data controllers may have to comply with legal requirements in ways that involve the processing of data. This could be for instance when they are ordered by the courts to hand over certain data or when they are forced to defend themselves by legal proceedings (including where started by the data subject). Whilst such a potential ground may have societal importance, it is hard to see its potential relevance in permitting scientific research.

#### Legitimate interests

One legal base that is likely to be of more relevance to research is that of ‘legitimate interests’.^26^ This represents an extremely broad option for non-governmental or public entities to process personal data when it is needed to do so in their interests (Donnelly and McDonagh 2019).^27^ Where available it means that consent will not be required for processing of sensitive data (Olly 2018). This could for example include any number of instances where large commercial entities may wish to conduct further research on their customers’ data so that they can improve services to them, reduce the risk of criminality or other harms or improve their general commercial strategy. The concept of legitimate interests does however have some important limitations, some of which are outlined in recital 47 of the regulation. It states:

*“The legitimate interests of a controller, including those of a controller to which the personal data may be disclosed, or of a third party, may provide a legal basis for processing, provided that the interests or the fundamental rights and freedoms of the data subject are not overriding, taking into consideration the reasonable expectations of data subjects based on their relationship with the controller. Such legitimate interest could exist for example where there is a relevant and appropriate relationship between the data subject and the controller in situations such as where the data subject is a client or in the service of the controller. At any rate, the existence of a legitimate interest would need careful assessment including whether a data subject can reasonably expect at the time and in the context of the collection of the personal data that processing for that purpose may take place.”*

The GDPR thus demonstrates that the relationship between the data controller and the data subject is central in determining how far the ground of legitimate interest can be used to justify processing of personal data inter alia for research purposes. Where the relationship is of greater proximity and transparency it will be easier for proposed research use of personal data to be foreseeable. Foreseeability (or ‘reasonable expectations’ as the GDPR describes) is crucial because it allows data subjects to exercise their data rights (e.g. the right to object or the right of erasure) in instances where they may want to stop forms of processing that are likely to be conducted under the guise of legitimate interests.^28^

Conversely, when the relationship between data subject and controller is more convoluted and less transparent, it will be more difficult to argue that the research use of personal data is foreseeable in terms of the legitimate interests of the data controller. The use of legitimate interests might be more acceptable for example when a social media giant conducts research with its clients’ data in order to find ways to prevent future account hacking. Research of this type is arguably foreseeable and even to be expected by the data subject. Research by an another organization which only had a transient link with certain data subjects and for purposes that had a limited connection the original relationship might however be difficult to justify. In addition, as the EDPS pointed out in its opinion on scientific research (A Preliminary Opinion on data protection and scientific research”, European Data Protection Supervisor 2020), data controllers must perform a balancing exercise when discerning what is permissible in terms of their legitimate interests. This test was previously outlined by the Article 29 working party in 2014.^29^ It is complex and involves balancing the legitimate interest sought by the data controller against the fundamental rights and freedoms of data subjects. This will involve taking into account the importance of the processing in question (to both the data controller and society at large) and balancing this against the interests of the data subject, including taking into account and potential privacy harms. Research that has a high value to the controller or to society in general will carry a higher weight in such a balancing exercise.

### B. Informational obligations/data subject rights may vary according to the legal base used

In discerning what requirements may be attached to a particular legal base it is important to take into account the informational obligations and data subject rights that may be applicable in each instance.

(i) *Informational obligations connected to the use of differing legal bases*

In terms of informational obligations, two GDPR articles are of particular importance. Article 13 outlines what information must be provided when data subjectd provide their consent for the processing in question. This includes information such as the name and contact details of the data controller, the aims of the processing and further information concerning the potential exercise of their data subjects rights. In general compliance with these requirements is not particularly difficult. This is because such information can be provided at the same time a data subject is providing consent (e.g. with a physical or electronic consent form). This is obviously not the case for the other legal bases described above where the data subject does not provide consent. In such instances article 14 comes into play, outlining certain forms of information that should be provided when consent has not been obtained from data subjects for the processing in question. Complying with article 14 in many instances of scientific research is however likely to be more problematic than may be the case for article 13. In many instances this may for the same reasons outlined above that make obtaining consent itself difficult. This may be because it may be difficult to contact all data subjects or systematically gain consent from them. Having to contact data subjects even in instances where consent is not used as the legal basis for processing would often present serious problems for researchers that opt for other bases such as public interest or legitimate interests (where such bases are indeed available). The GDPR appears to recognize that this may be a problem in inter alia research stating that the requirements of article 14 do not need to be met where^30^:

*“the provision of such information proves impossible or would involve a disproportionate effort, in particular for processing for archiving purposes in the public interest, scientific or historical research purposes or statistical purposes, subject to the conditions and safeguards referred to in Article 89*(1) *or in so far as the obligation referred to in paragraph 1 of this Article is likely to render impossible or seriously impair the achievement of the objectives of that processing.”*

This exception seemingly applies different tests to different research contexts, including the legal base that is opted for. For public interest or scientific research, the bar appears to be the creation of ‘disproportionate effort’. For other forms of processing it appears to be set higher i.e. ‘to render impossible or seriously impair’. This is important given that all of the legal bases discussed above may be used for research. Accordingly research that uses legitimate interest as a base will face a higher bar to demonstrate that they can use the exception outlined in Article 14 to not provide information to research subjects (i.e. they will need to demonstrate serious impairment or impossibility). Difficulty or high cost would not seem to be valid reasons. As Ducato states (Ducato 2020):^31^

*“This is not in any case a blanket exception and requires a balancing assessment. First of all, the “impossibility” and “disproportionate effort” must be tailored to “the number of data subjects, the age of the data and any appropriate safeguards”. Second, the EDPB has further stressed the need for the controller to evaluate “the effort involved for the data controller to provide the information to the data subject against the impact and effects on the data subject if he or she was not provided with the information”. This will consist at least of making the information publicly available (e.g. publication on website, newspapers, etc.)”*

The room accorded to data controllers (including to researchers) by article 14(5) should thus not be overestimated. The author would argue that this will be particularly the case for research by private entities under the guise of legitimate interests. In situations where the ‘legitimate interests’ base are most likely to be applicable, i.e. where the data subject has reasonable expectations that research with their data may occur there will more often than not be a link between data controller and subject (e.g. customer/client) that will arguably make it difficult to claim that providing the information outlined in article 14 of the GDPR will render the research in question possible. Where this is the case the advantages of using legitimate interests as a legal base over consent will, to a large extent, be negated (given the need to provide sufficient information to the data subject).

(ii) *Data Subject Rights may vary with certain legal bases*

A somewhat similar situation also applies in terms of data subject rights. This is because article 89 of the GDPR permits member states to limit the application of data subject rights where research is for public interest or scientific research purposes (Article 89(2) states “Where personal data are processed for scientific or historical research purposes or statistical purposes, Union or Member State law may provide for derogations from the rights referred to in Articles 15, 16, 18 and 21 subject to the conditions and safeguards referred to in paragraph 1 of this Article in so far as such rights are likely to render impossible or seriously impair the achievement of the specific purposes, and such derogations are necessary for the fulfilment of those purposes.^32^ In particular this relates to the rights to access, rectify, restrict and object to the data processing in question.^33^ Facilitating each of these rights may create difficulties and burdens for researchers, depending on the particular research context in question. This may entail structuring some research differently or even making some research more expensive. Compliance will likely entail the need to for important administrative infrastructures and measures that will allow researchers to comply with data subject requests (Quinn and Quinn 2018).^34^

Given this, the ability to use a legal base where such rights have been restricted may be an important advantage. The GDPR allows Member States to restrict the availability of such rights where processing is for “scientific or historical research purposes or statistical purposes”.^35^ The choice of words here is interesting because it seemingly corresponds with the description of the legal bases outlined in article 9(2) (discussed further below in section 5) or potentially the option for further processing for scientific research (discussed in (C.) directly below). This appears to indicate that that this ability only applies where one of these options are chosen and not others such as ‘consent’ or ‘legitimate interests’. In terms of the former this is logical given the importance of data subject rights - it would surely be inappropriate if they could be restricted in too wide a manner. Given this, the need for Member State legislation, that would only apply in specific contexts is important (for more see the discussion in section 4.A) because such legislation will only apply in certain circumstances where the limitation of such key rights could be considered appropriate.^36^ In many Member States such legislation may for instance only be applicable where research is perceived as being in the public interest (Dove and Chen 2019). Another important factor is that article 89 only appears to offer such limitations to be possible when “appropriate safeguards” are in place. In the research context this arguably relates to adequate forms of ethical governance, something that public sector research institutions are more likely to have in place than those in the private sector. Given these factors, it can be considered that public sector research organizations are more likely to be able to benefit from the ability to relax data subject rights than commercial entities. As discussed below however the second potential option of a valid legal base existing simply for ‘further processing for scientific research’ is more puzzling and raises serious questions about the potential limitation of data subject rights in a wide range of contexts.

### C. The possibility of ‘further processing’ for scientific research

In addition to the legal bases outlined the above the GDPR contains one extremely broad an important provision that should be considered alongside the availability of legal bases that could be used for scientific research. This relates to article 5(1)(b) which states that personal data must be:

“collected for specified, explicit and legitimate purposes and not further processed in a manner that is incompatible with those purposes; further processing for archiving purposes in the public interest, scientific or historical research purposes or statistical purposes shall, in accordance with Article 89(1), not be considered to be incompatible with the initial purposes (‘purpose limitation’)”

This provision is remarkable in its potential breadth. Unlike many of the elements of article 6 discussed in part (a) above, it seemingly does not create a separate legal base for the processing of personal data. Rather, it appears to indicate that if a data controller has a valid legal basis for possessing personal data in the first place, it can subsequently process the data in question for purposes of scientific research. It can arguably be interpreted in one of two ways. The first is that no legal basis is required for the processing of personal data for the purposes of scientific research as long as there was a prior legal base for the collection of the data in the first place. The second is that the original legal base is still valid for such forms of processing, even where the legal base itself and the original context of data collection seemingly had little to do with scientific research. The first would be perhaps the most conceptually remarkable because it departs from the general approach within the GDPR that a specific legal basis is always required for any form of processing. Doubt may be cast over this interpretation given that the drafters of the GDPR did not opt make a legal basis in article 6 of ‘further processing for scientific research’ (i.e. one that does not require member state legislation).

The practical ramifications of this provision are however likely to be even more important that the conceptual confusion it creates. An expansive reading would seemingly indicate that a data controller that is legally in possession of personal data can carry out scientific research with it. This seemingly includes an almost indescribably wide range of contexts throughout both the public and private sectors. In terms of the latter for example the frequency and variety of contacts that individuals have with various commercial entities mean that their personal data could be used for a potentially enormous range of processing activity that could be termed as scientific research. In discerning just how broad this possibility might be it is important to consider the broad understanding of the term scientific research which the GDPR calls for (discussed in sections 3.A). This view of what can constitute scientific research means that many forms of processing that might not have been traditionally thought of as scientific research may be categorised as such by the GDPR. This includes for example efforts at product and service innovation by private enterprises, something that in the modern world could take an almost indescribable variety of forms.

It must be acknowledged however that article 5(1)(b) does contain some limiting factors that will serve to restrict its use to a certain extent. This includes references to processing “in accordance with article 89(1)”. As discussed this entails the need to have the necessary organisational measures (including potential systems of ethical review). Such structures may entail financial and administrative costs and may also impose additional requirements on the desired research in question. Whilst these may pose major difficulties for smaller entities, it is likely that larger financial entities for example will be able to put into place such measures (though doubt may be raised about their integrity and transparency) in many instances that will be seen as capable of demonstrating compliance with article article 89(1). This may for instance include ethical policies and procedures for review of processing activities.

Another important limiting factor is the need to comply with the informational requirements of Article 14 of the GDPR (as discussed in section 3.B above). This relates to a requirement to impart information about the processing activities to the data subjects concerned. This inter alia allows data subjects to invoke their data subject rights should they wish to do so (including potentially the right to be forgotten).^37^ Whist this may form an important impediment in a number of contexts (where efforts must be made to contact various data subjects) this burden is significantly eased by the extra room permitted to scientific researchers by the GDPR in interpreting this requirement. As section 4.B discusses this requirement can be dispensed with when it requires a ‘disproportionate effort’.

Despite this limitations however the possibilities provided by article 5(1)(b) are enormous and are poorly clarified by the regulation itself.. A simple understanding of the wording used would seem to indicate that any instance of legal processing of personal data will give an entire range of entities the problems to conduct a variety of further processing under the umbrella of scientific research. Given the wide vision the GDPR seems to have of what can constitute scientific research it could be argued that this potential breath of this provision raises some serious concerns. This could for example include a wide spectrum of processing, much of which may only be loosely associated with conventional notions of scientific research (i.e. to include product innovation and better customer targeting/service delivery). Given that many commercial entities already legally possess an enormous range of their clients’ data, article 5(1)(b) seemingly provides them with an enormous potential to further use it under the guise of scientific research. Given such concerns, it is unfortunate that the EDPS chose not to deliver further commentary in its recent opinion on scientific processing.^38^ It did however state that such commentary would be forthcoming in the future.

## 5. Potential legal bases for research with sensitive data

### A. Many forms of research may use sensitive data

The GDPR foresees a special regime for sensitive data (termed ‘special categories’ of data). This includes a different set of legal bases for controllers that wish to process such forms of data. For researchers it is important to be aware of the existence of sensitive data and that different conditions may apply to their processing than other forms of personal data. Such data is defined by the GDPR as:

*“personal data revealing racial or ethnic origin, political opinions, religious or philosophical beliefs, or trade union membership, and the processing of genetic data, biometric data for the purpose of uniquely identifying a natural person, data concerning health or data concerning a natural person's sex life or sexual orientation shall”*

The breath of the definition of sensitive data is important. Not only are each of the elements described in article 9 of enormous potential breadth, many are likely to include forms of data that are of immense, social, societal and economic importance. They are therefore likely to be of interest to researchers (Quinn and Malgeri forthcoming-a).^39^ Health data is an important example and provides a good illustration of the potential breadth of sensitive data. The concept of health data itself goes far beyond medical data or medical dossiers and has been argued (by the article 29 working party) to include all personal data that is capable of providing an indication of the health status of specific individuals. This includes not only data relating to illness or health problems that an individual might have, but also data that provides an indication that an individual will develop a health problem in the future and even information that indicates a specific individual is simply ‘healthy’.^40^ Given that various pieces of information that individually say nothing about an individual’s health status can be combined to provide such an indication, many forms of big data may be health data (often without being that being readily apparent). As a consequence, many forms of data that are sought after by researchers are likely to contain health data, whether researchers are aware of this or not. This goes far beyond traditional (and more obvious sources of health data) such as official medical records and can include (less obvious sources of health data) questionnaires, to information about movement (including from GPS mobile data) and social media. Data from such sources can be combined with increasing ease (due to increased computer power, the increased availability of complimentary forms of (often big) data and ever more powerful analytical software (often using artificial intelligence) to reveal information about the health status of specific individuals (Article 29 Working Party Opinion on Anonymisation Techniques 2014). An analogous form of reasoning can also be applied to the other forms of sensitive data described in article 9 of the GDPR (Quinn and Malgeri forthcoming-b). In most cases (excluding genetic and biometric data), various elements that might not be themselves sensitive in nature, can be combined to provide inferences that may be of a sensitive nature. As a result of these factors, the proportion of data that researchers use in the future that is of a sensitive nature is only likely to increase.

The use of sensitive data brings with it a number of extra burdens. This includes the likely need to appoint a Data Protection Officer (DPO) and perform a Data Protection Impact Assessment (DPIA) (Kloza et al. 2017).^41^ Each of these will impose added administrative burdens on those who wish to process sensitive data, including for research purposes (Quinn and Quinn 2018).^42^ Whilst these are important requirements that should not be underestimated, they will not be explored further in this article given they apply to most forms of research with sensitive data irrespective of the identity of the data controller or the legal base used. Again, as with non-sensitive data, the remaining focus will be on the legal bases available to researchers given that they may in reality be differently available to different types of actors engaged in research.

### B. A number of important legal bases are relevant for research with sensitive data

There are a number of important bases that are likely to be relevant for research with sensitive data. As with bases for non sensitive data, the reality is that some of these grounds are more likely to be available to some actors than others. The most important grounds for research are:

#### Explicit consent

As for non-sensitive personal data, consent is usually considered as one of the most important bases for the use of sensitive data (Corrigan 2003). Not only is the legal underpinning of this base relatively easy to understand, it is often considered the most ethically favorable option where it is possible (Mostert et al. 2016). For this reason, the use of consent as a legal base is often considered as the default options by researchers. Ethics committees will often expect it to be used where it is possible. The existence of two forms of consent (one for sensitive data one for non-sensitive data) has been a feature of the European data protection framework since Directive 95/46/EC (Quinn et al. 2013).^43^ The directive foresaw a looser form of implicit consent for non-sensitive personal data and a more demanding form of ‘explicit consent’ for the use of sensitive data. In areas such as health care, such a division was important and was often understood in national law as requiring signed, written consent. The GDPR has however ‘muddied the water’ in terms of the difference between explicit and non-explicit consent. In particular, it has strengthened the requirements around consent for non-sensitive data, essentially making implied consent impossible. In place of this an ‘unambiguous indication’ is required.^44^ At the same time, the GDPR makes clear that explicit consent need not be written, though a record that such consent has been given must be kept.^45^ The difference that remains is seemingly the requirement that data subjects acknowledge they are giving explicit consent, i.e. that a clear recognition that a formal act of consent is being given. This will usually entail explicit acknowledgement by the data subject that consent is being given. As with the use of consent as a legal base for the processing of non-sensitive data, one of the most important benefits of using consent as a legal base for the processing of sensitive data is its versatility. As a legal base it is not intended to be applicable only to a specific context or setting but is extremely versatile, capable of being applicable to numerous contexts, including scientific research. Similarly, it is available to all the various types of entities that might want to conduct research throughout both the public and private spheres (and everything in between). This is not the case for many of the other legal bases that can be used to process sensitive data for research, which may be more or less suited to certain types of actors (discussed further below).

#### The scientific research exception

As with consent for the processing of non sensitive data (see section 4), the use of ‘explicit consent’ for research problems may raise serious issues in some contexts (Corrigan 2003). Unlike non-sensitive data which sees a general base for processing in the public interest, the GDPR foresees a series of more precise (at least in a thematic sense) legal bases that relate to specific contexts for the processing of sensitive data. One of these is for the “purposes of archiving or scientific research” (Donnelly and McDonagh 2019). In the literature this if often termed ‘the scientific research exception’ as it is often viewed as an alternative to the basic presumption that explicit consent is the default legal base to be used for research purposes involving sensitive data (Carter et al. 2015).^46^ As with the use of the public interest legal base for non sensitive data (see section 4), there is however important conditionality attached (Quinn 2017; Hallinan and Friedewald 2015).^47^,^48^ Again, the use of such a base should be outlined in Member State or Union law and be both necessary and proportional. This means there should be a valid reason for not using other bases such as explicit consent. In most cases relevant legislation should outline what type of actors can make use of this legal base and in which circumstances. Sometimes such legislation can grant a broad discretion to certain entities (e.g. universities), whilst in other instances it may focus only on specific actors (e.g. certain governmental bodies) (Taylor et al. 2018). Given the need for legislation and all of the complexities surrounding it, it is more likely that such an option be available for large public entities (e.g. universities, health care actors) than for private entities. As section 6 discusses, the availability of the scientific research exception can vary between countries, with certain Member States seemingly more willing to let commercial entities avail themselves of this possibility (though it may still be harder for them to do so than it is for public sector entities).

#### Public health /substantial public interest

In addition to the scientific research exception there exist other bases that, although are not explicitly directed towards research, could in appropriate circumstances be used to conduct research in certain contexts. This includes for reasons of ‘public health’ and ‘substantial public interest’.^49^ Again, as, with the scientific research base, these bases allow the processing of sensitive data without consent where there is specific legislation in national or EU law allowing so. This may, as section 4 discussed be necessary in contexts where consent is clearly inappropriate because of imbalanced power relations. Although not specifically intended to deal with research, it is possible that these legal bases can be used to cover research when it is intended to meet an aim falling within this area.^50^ This could for example include research into numerous public health issues, ranging from issues associated with obesity, to the spread of pandemics (including currently with COVID-19). The ability to conduct such forms of research is important in inter alia allowing governments and public authorities to respond to serious challenges that can arise. Once again, the requirement for existing legislation specifying when such processing can occur will significantly restrict the types of entities that can avail themselves of this option. It should also be noted that the terms ‘substantial public interest’ outlined for sensitive data denotes a significantly higher threshold than ‘in the public interest’ (which is available for the processing of non-sensitive data). This will restrict the availability of this legal base further. This may for example mean that it may not be considered a suitable base for forms of blue skies research where the direct benefit in terms of public interest may not be immediately clear (Hallinan 2020).^51^ The same is likely to be true for research that has a primarily commercial motivation.

#### Data that is manifestly made public

One controversial ground for processing sensitive data relates to sensitive data that has been “manifestly made public”. This base is particularly striking given that it is not available for the processing of non-sensitive data which is arguably of a far higher risk. Unfortunately the GDPR does not clearly elaborate the limits of this legal ground. The European Data protection Supervisor has recently provided guidance on when this base can be used stating:

*“Special categories of data may be processed if the data subject has manifestly made them public. EU data protection authorities have argued that this provision has to be ‘interpreted to imply that the data subject was aware that the respective data will be publicly available which means to everyone’ including, in this case, researchers, and that, ‘In case of doubt, a narrow interpretation should be applied, as the assumption is that the data subject has voluntarily given up the special protection for sensitive data by making them available to the public including authorities’. Publishing personal data in a biography or an article in the press is not the same as posting a message on a social media page.”*^52^

Whilst this clarification is not extensive, it confirms two important things. The first is that sensitive data can indeed be processed when it has been made manifestly public by the data subject. This clarification is important given the ambiguity discussed above (i.e. that a similar base is not available for non-sensitive data). The second is that the provision should be interpreted with care in an extremely context dependent manner. The most important issue is one of awareness and whether a data subject can have expected that a particular use was made of his or her data. This question of awareness is complex in the context of research. One might ask in which situations individuals make their details available in the genuine understanding both that they are made public and that they may be used for research purposes? Is it realistic for example to expect individuals to realise that when they place their data on a web site it may be harvested by complex web crawling operations, packaged into large big data sets and subjected to research (with a diverse range of motives) with various forms of novel AI based techniques? (Massimo 2016) Whilst the answer may be yes for some individuals who have specific awareness of these areas, for most people the answer will likely be no. It is important to remember that given modern computing power, and the interconnectedness of the online world, the risks of harms occurring with research on sensitive data may be difficult to envisage, especially in the long term. Imagine for instance genetic data that is published online. The variety of likely unknown developments in the science of genetics make it extremely difficult to predict the nature and scope of future research (Quinn and Quinn 2018).^53^ Given this, the author would argue that, unless made public in areas where awareness of research was indeed evident (e.g. particular online fora, websites communities etc.) it would not be prudent to assume ‘awareness’ of potential research (and especially complex research) in a way that the EDPS appears to be demanding.^54^ A better approach would be to only extract data in instances where it had been accompanied by some sort of explicit definition of ‘public availability’, including importantly in the context of this paper, for research purposes. This will however have the obvious effect of limiting the potential use of this legal base.

## 6. An unlevel playing field?

The forging analysis demonstrates the wealth of options that the GDPR provides for research. The regulation can not be considered hostile to research, nor does it attempt to promote a ‘one size fits all’ approach to all forms of research. Its drafters clearly intended to provide opportunities for various actors, in various contexts to conduct research when certain forms of conditionality are met. This raises the questions as to whether various types of actors enjoy a level playing field in terms of their ability to conduct research with personal (including potentially sensitive) data. This question is important because the world of research is not populated by uniform actors operating in similar contexts but rather, a diverse array of actors operating in vastly different contexts. Innovative and socially useful research is not the domain of one particular entity or class of actor but can be conducted by a variety of entities (Hartley et al. 2017). This can vary from the traditional university research department, to medical institutions, to small business to large and enormously powerful commercial entities (Maroto et al. 2016). Each of these operates in different environments, with access to varying levels of resources and possessing different abilities to access personal data. Some of these are described below.

### A. Universities

(i) *Consent as a default base that is not always appropriate*

Universities represent the classic image of a research institution. Often publicly funded (or at least heavily subsidized) they are usually perceived (erroneously in some cases) as carrying out research not primarily because of a commercial interest but to advance scientific understanding or to address an important public interest need. Given the nature of their work they may be expected to undertake research that would not otherwise occur in the private sector. Such research may be unlikely to deliver an immediate financial return (Watts 2016).

Consent is the most important legal base for research with personal data in universities (Mostert et al. 2016). Its versatility allows it to be deployed in vastly different contexts with different types of research subjects and research of vastly differing aims. Universities as mature research institutions often have well developed and complex systems of ethical review (Vadeboncoeur et al. 2016; Kohn and Shore 2017). Such processes may be required by a university’s articles of establishment, demanded by funding bodies or obliged by national legislation. Most research carried out involving human participants or the use of personal data will be expected to gain approval by a university ethics body before commencement. In most instances, researchers at such institutions will depart from the expectation that where possible consent should be used as the legal basis for research using personal data. As the author has discussed in a previous paper, ethics bodies may push this attitude (sometimes too aggressively) where consent may not be appropriate. This may on occasion force a ‘consent or anonymize dilemma’ on researchers (Quinn 2017).

Although consent is useful, and in many cases clearly the preferable choice of legal base for ethical or practical reasons it is in many occasions not suitable (Donnelly and McDonagh 2019). As with other forms of research, universities are increasingly using large and complex forms of data. This may involve collecting or further processing personal data that relates to numerous data subjects. Assembling the types of large and often heterogeneous data needed for many forms of modern research may present a range of practical, ethical and legal problems, particularly where consent is used as a legal base. As section 4 discusses, in research with many data subjects it may be practically difficult to physically contact all research participants or gather them together in a way that is possible to obtain informed consent. Even where it is, problems with power relations or vulnerable data subjects (e.g. children) may render consent ethically undesirable. In addition, university researchers may want to reuse data for subsequent analysis, in a way that is materially different than the initial research that they conducted (Mcguire et al. 2008). In such cases, re-obtaining consent for all data subjects may be a disruptive exercise. The availability of the a legal base that does not require consent is thus important for many forms of research.

(ii) *The importance of the scientific research exception*

Legislation exits in all EU Member States that permits universities to process both non sensitive and sensitive personal data for aims of scientific research (Molnár-Gábor 2018).^55^ This allows universities to process under the public interest or scientific research grounds described in sections 4 and 5 respectively. Such legislation can be broad, not referring to specific forms of personal data or it may refer to specific types of personal data “e.g. genetic data” (Taylor et al. 2018). Such legislation can thus in theory present an extremely broad ground for the universities to base their research upon. Whilst the availability of such legislation is a welcome and broad ground for researchers, using it is often not so simple. This is because university researchers will usually have to obtain ethical approval and explain why they are not using consent as a legal basis. As section 5 discussed, some ethics bodies may be more hostile than others concerning the use of the scientific research exception (or more specifically the processing of personal data in the absence of consent) (Mostert et al. 2016). Ethics bodies are for a number of reasons (many of which are correct) likely to greatly privilege research that is able to gain the consent of data subjects. This may lead to situations where researchers are not able to use the scientific research exception found within the GDPR, even though consent may not be suitable. In other instances they may be pressured into gaining some form of consent even where the scientific research exception is used (in such cases the use of consent represents an exercise in ethical compliance and does not need to meet the requirements of consent as a legal base as laid out in the GDPR).^56^ Such factors can arguably lead to certain forms of research being disincentivized (i.e. where gaining any form of consent is truly problematic) and mean that in reality the availability of the scientific research exception may be less than that which would be apparent from a simple reading the GDPR itself.

### B. Other public bodies

As with universities, a large variety of other public organizations may have to conduct research on personal data for a variety of reasons. This could range from public health bodies that attempt to model and predict the scale of potential or impending epidemics or other public health threats to bodies intended to regulate health and safety in the work place (Srncova et al. 2019). Research may be important in developing new strategies to safeguard the public from various existing or potential future harms. An obvious example is readily available in the ongoing COVID-19 pandemic (Malgeri 2020). In other cases it may be difficult to draw the line between research and important managerial activities, including the organisation of public services (Klievink et al. 2017). Conducting such activities inevitably requires forms of research that will, on certain occasions require the processing of personal data. In many cases consent may not be feasible for many of the reasons discussed above in section 4.^57^ As section 4 & 5 describe, the GDPR accordingly foresees a number of legal bases that are more likely to be applicable. These include (as with universities) the public interest base (for non-sensitive data), the public health, substantial public interest and scientific research bases (for sensitive data).^58^ In such instances where legislation outlines that a particular public body can conduct certain forms of research using personal data it can do so without consent. Depending on the body in question, it may or may not have ethical review procedures in place. In some cases ethical or deontological codes make exist that provide guidance or complaint mechanisms may exist to hear from individuals that feel that an incorrect course of action has been taken. Whilst providing an important form of redress, such procedures may not be as systematic or exert as high a level of scrutiny as usually occurs with university ethical review.

### C. Commercial entities

Private entities (with some prominent philanthropic exceptions), will usually conduct research because of commercial or financial motivations. They may do this to be able to better deliver products to their customers, to better understand their tastes and desires and to be able target them with advertising more correctly (Moore and Tambini 2018). The role of large commercial entities in research is going to become ever more important in the future given the enormous amounts of data they are able to gather. Tech giants and social media companies (e.g. Google, Amazon and Facebook) are able to gather and store enormous quantities of customer data on an ongoing basis (Sharon 2016). This provides an incredible pool of data upon which such entities can perform research for a variety of reasons. Indeed, it is their ability to access and utilize such enormous amounts of data for innovative purposes that has largely been responsible for the atmospheric increase in their value as commercial entities (Klievink et al. 2017). This ability has stemmed from the range of complex services these companies are able to offer a large range of clients on a global basis. Such organizations are thus able to ‘harvest data in a manner and on a scale that university research groups can in general only dream of. This ability is only likely to increase further in the future as a consequence of increasing technological progression and the consequent generation of data (inter alia through developments such as IoT).

In terms of legal bases, the picture for commercial entities may be somewhat different. Whilst they will also have the default option of using consent as a base for processing, the availability of some of the other options described above may be limited. This is not because the GDPR makes a distinction about such bases being available for only public sector bodies, but more to do with the practical realties of facilitating the use of these legal bases. This is in particular related to the need for law outlining who can do what type of processing (where for instance the scientific research exception is used). Whilst large and important public sector entities such as universities, university hospitals and large research institutions may have been considered important enough to merit legislation facilitating the use of a personal data for research, this may not be the case for commercial entities (Taylor et al. 2018).^59^ The chances that specific laws will be made facilitating the use of public interest bases by commercial organizations is small. In most cases, the interests of large commercial entities will not be seen as being in the public interest, and attempting to make the case that it is would likely come at a political price (For an alternative view see the report composed by (EPRS) - European Parliamentary Research Service Scientific Foresight Unit (STOA) 2019).^60^ This is especially true in an age where the suspicion of the power and reach of such entities is growing stronger. As a result, and even in cases where a large commercial entity may claim to want to act in the public interest in a philanthropic way, it is unlikely that legislation will be created to facilitate processing of personal data without consent. For smaller commercial entities the creation of such legislation is even less likely given that the necessary legislative time is not likely to be accorded in order to facilitate the activities of smaller organization. Where such specific legislation is not available it may often be difficult for commercial entities to rely on exceptions that have been crafted primarily with the public sector in mind. This is particularly true for the ‘public interest’ based exceptions including the ‘scientific research’ exception available for sensitive data. Some states (e.g. the UK) (ICO report n.d.)^61^ only allow its use where it is demonstrably in the public interest. Whilst it is of course possible that some forms of commercially motivated forms of scientific research will be in the public interest (e.g. pharmaceutical research), it is likely that most forms will not be able to meet such a test. Others may even require approval of specialist committees (e.g. Ireland) (Dove and Chen 2019).^62^ Whilst there are certain exceptions, the general rule of thumb is that it will be more difficulty for private or commercial entities to avail themselves of ‘the research exception’ outlined in the GDPR.

As section 4 discussed however, commercial entities have a number of other legal bases available to them that may not be so readily available to the public sector. These include research performed in order to ‘fulfill contract obligations’ which may be relevant where individuals have contracted with a commercial entity directly to perform research or for other services where research may be clearly implicit.^63^ More important however is the potential use of the ‘legitimate interest’ grounds and the possibility for ‘further processing for scientific research (discussed in section 4.C).

Unlike the public interest based options, legitimate interests is not permitted as a legal basis for public entities and is available only for private sector data controllers.^64^ As discussed in section 4 this basis permits an enormous range of processing that will likely encompass many forms of research. Its use is limited by the need to conduct a balancing exercise, taking into account the interests of data subjects and weighing it against the legitimate interest sought by the data controller. Central to deciding whether such a balance supports a particular instance of processing is how foreseeable the type of processing is on the part of the data subject. Where it is clear that such a form of processing can be expected it will be easier to argue that the balancing exercise supports processing in the legitimate interests of the data controller. The logic to this is that where such a form of processing is foreseeable (or within the ‘legitimate expectations’ of data subject as the Article 29 Working Party described it),^65^ individuals can choose not to provide data to the data controller in question or, where the data controller is already in possession of it, exercise their rights as a data subject to prevent the type of processing in question (this may include exercising one’s right to object or have their data deleted). Accordingly, the GDPR makes it clear that where there is a clear relationship between the two parties (e.g. customer and client) the chances will be greater that a particular form of processing will be foreseeable. By extension, where data controllers make such activities known to their clients (e.g. through privacy polices) it will be easier to argue that processing under the guise of ‘legitimate interests’ is permissible. Where such a relationship exists, it will also be easier for data controllers to comply with their requirements *vis-à-vis* article 14 GDPR. As section 4 discussed, these relate to the details of the processing operation that should be provided to data subjects when their data has not be obtained through consent.

In terms of the second option described above, i.e. ‘further processing for scientific research’ the scope of use may even be wider. This potential possibility is less well defined, either in the GDPR or in any form of expert guidance that has been given under its authority. Even if its use is to be subject to further (as of yet not clear) restrictions, its potential for use may be extremely significant for similar reasons to that of ‘legitimate interests’ (i.e.; given the potential amount of data that many entities have) and also the potential flexibility of the concept of ‘scientific research’ under the GDPR. There are some subtle differences however. On the one hand, some of the restrictions applicable to the use of ‘legitimate interests’ base are seemingly not applicable to the ‘further processing’ option. This includes requirements to demonstrate legitimate expectations on the part of the data controller (including for example a relationship of proximity). On the other hand, the further processing option, unlike legitimate interests’ is subject to the requirement of compliance with article 89 (i.e. the need to create organizational structures to safeguard the rights of data subjects – discussed in section 4.C.). Notwithstanding the urgent need for further clarification of the extent of this processing option, it is clear that its availability alongside the ‘legitimate interests’ provides enormous possibilities for many types of actor, particularly in the research sector.

Given that large tech and social media giants have hundreds of millions of users with which they often have a customer-client relationship (and often with carefully crafted privacy policies), they can be argued to possess unparalleled pools of data upon which they may be able to perform research under the guise of ‘legitimate interests’ or ‘further processing for scientific research’. Firms such as Amazon have evolved and expanded continuously based upon innovate commercial research carried out on customer data. This has allowed the company inter alia to better target particular products based on its customers online behavior. This provides such entities with a source of data upon which they can perform research that is often simply not available for most universities. With the exception of university hospitals and their relationship with patients, most universities do not have vast repositories of client data available. By contrast, they often have to request data from external entities and may thus be greatly beholden to their willingness to co-operate. This is an important de facto limitation that acts to lessen the seemingly wide latitude granted by the GDPR’s research exemption.

## 7. Are private sector interests favoured by the GDPR?

The discussion above raises questions as to whether private sector researchers enjoy an advantage over their public sector equivalents. In particular one might ask whether the ability of private entities to use legitimate interests trumps public sector research’s likelier easier access to the GDPR’s ‘research exception’? Similarly, whist not only available to private sector interests, one might ask whether the possibility of ‘further processing for scientific research’ is more likely to be available to private sector interests in many instances? In asking these question it is necessary to take into account the de facto context researchers are likely to find themselves in. The situation of considerable data availability for large private entities can be contrasted with public sector research institutions, which may often not have direct access to the data they need for research (this is particularly true for universities). There is nothing in the GDPR to compel external entities to hand over data that may be useful for research, even where its processing may be legitimate for purposes of scientific research. External entities may refuse because of perceived commercial interests, or because they must submit such decisions to their own form of ethical oversight (e.g. hospitals) (Markoff 2012).^66^ An increasing dependency on big research data arguably makes research institutions such as universities more dependent on external big datasets. Researchers may find it difficult or impossible to assemble these alone and are likely to become increasingly dependent on agreements with external entities to provide data that is necessary for research (Hao 2020).^67^ In this modern big data context, the possibility to process personal data under the GDPR is only one part of the overall picture. Having the theoretical legal possibility to process such data for research ends arguably means very little if researchers have no access to the data in the first place.

This situation described above can be contrasted with the situation that commercial entities find themselves in. Whilst they may be limited by the GDPR or Member State legislation in terms of their ability to take personal data from disparate sources, for which they have a limited connection or relationship with the data subject, this may not be a serious problem when the controller in question is in possession of large amounts of data concerning individuals with which it does have such relationship. This may be the case where particular data controllers have large bases of customers to whom they provide goods and/or services to. As section 6 discusses, large online entities that sell goods or provide online services (e.g. social media) are likely to be in such a position. Such actors may as a result legitimately have access to heterogenous forms of data from numerous data subjects spread over many locations. The research value of such data may be immense and is only likely to increase further given never ending augmentation of both the volume and variety of the data in question.

The factors above could arguably seem to indicate a real de facto advantage in the ability of private sector entities to conduct research the era of online connectivity and big data. The sheer amount of data that many large commercial actors may possess, taken together with the existence of a legal grounds such as ‘legitimate interests’ or ‘further processing for scientific research’ and the relative weakness of their forms of ethical review may often provide an advantage in terms of the ability to conduct research, particularly with big data. It is important however to remember however that these grounds are not available for the processing of sensitive data (for research or any other purposes).^68^ The reason for this is that they are not listed in Article 9. This is unlike the major grounds commonly used by public sector actors which are outlined both in Article 6 (for non sensitive data) and Article 9. The effect of this means legitimate interests can only be used as a legal basis for processing sensitive data if it can be combined with a legal basis outlined in article 9 and which is clearly applicable.^69^ The result is that without being able to depend on another basis for processing sensitive data, legitimate interests/further processing for scientific research alone will not be sufficient. This factor renders the available advantage to private sector researchers of these options less significant than it might seem, especially given the importance of sensitive data to many forms of research.

This creates an extremely important limitation to the advantages private sector entities may possess in terms of their research potential when compared to public sector entities such as universities. This is because whilst they may have a certain de facto advantage in their ability to conduct research with non-sensitive data, this advantage disappears with regards to sensitive data. Given the wide ranging description of sensitive data (outlined in section 4), this is a considerable disadvantage. Forms of sensitive data are often those that are the most interesting to researchers, especially in the type of research for which there is the most societal/economic demand (Quinn and Quinn 2018). Health data for instance is of immeasurable importance to many forms of research. In addition, the increasing importance of big data to modern research, which is becoming increasingly computational in nature means that more and more research will in general be using sensitive data. This is because as section 5 identifies, it is becoming increasingly likely that many forms of big research data will contain sensitive data due the possibility to draw sensitive inferences by combining various elements within it (even where this might not be intuitively obvious) (Quinn and Malgeri forthcoming-a). A reduced ability to process sensitive research data (without resorting to consent as a legal base) is therefore an important de facto impediment to research in the private sector that should not be underestimated. This places a severe limitation on the ability of private sector researchers to be able to rely on ‘legitimate interests’ as a base in the future, especially in areas of highly valued research (that often require sensitive data).

## Conclusion

The aim of this paper has been to discern to what extent there is a level playing field for both private and public based researche entities in terms of their abilities to use the legal bases made available in the GDPR for research. The reality is a nuanced picture. As this paper shows, it is necessary not only to look at a particular base as it exists on paper, but to envisage how it is likely to be used in reality. This is important because even if a legal base is capable of being used for research purposes in theory the reality may, depending upon the context in question, be very different. This may be particularly true if one compares public research institutions (e.g. universities) to private commercial entities where different types of actor may be able to make more use of the legal bases within the GDPR that can be used for research.

Whilst (assuming there is no serious imbalance in power relations) consent as a legal base is available to all types of entities wishing to conduct research (i.e. both public and private), the same may not be true for other legal bases that are important for research. This incudes bases for processing in the public interest (non sensitive data) and for scientific research (sensitive data). Both of these can be used to justify processing personal data for research purposes in various contexts. Both are in theory open to public and private research institutions alike. This appearance of equal opportunity however does not hold up to scrutiny in the real world context. This largely because the GDPR mandates that the use of such bases must be further clarified in Union or national law. In reality, this often translates into a need for national legislation outlining what types of entities can use these legal bases and in which contexts. Such legislation is important in allowing universities and other public research bodies to conduct research where for instance obtaining consent may not be viable (or desirable). The extent to which such a possibility is available to private sector actors depending on the wording of a particular Member State’s law. In many instances it might not be suitable for the type of ‘research activities’ that private entities often conduct. Such legislation may for instance specify certain types of entities (e.g. public sector bodies) that can avail themselves of a research exemption. Others may require that research be considered in the ‘public interest’, something not necessarily true of much private sector research.

Whilst private sector research may be disadvantaged in terms of its ability to utilise these bases, it has others which are not available to researchers acting in the public sector. This most notably includes the ground of ‘legitimate interest’. This allows data controllers to process personal data (including for research purposes) when it is in their interest to do so. This can only be done however where such processing is foreseeable for the data subject and it is necessary and proportional taking into account the interests of the data subjects. Whilst this does not provide private entities with a *carte blanche* to take personal data from anywhere and use it for research; it does mean that in many cases where such entities have a large base of customers or clients that they will be able to use their data for research. This includes instances where the motivation behind the research is primarily commercial in nature. Entities such as tech and social media companies that dispose of enormous and growing quantities of customer data will therefore possess enormous pools of big data that are very high in research potential. This can be contrasted many public research actors that are dependent on the cooperation of external parties to obtain research data.

Another important factor that should not be underestimated is that private entities are unlikely to have as rigorous procedures of ethical review as public research entities. Ethical review committees are unlikely to have as strong an institutional underpinning in the former type of organisation. Where they do exist such procedures are unlikely to be as transparent as they are in their public sector counterparts. In entities such as universities, ethics committees have an extremely powerful role in deciding whether research can go ahead. They will often heavily scrutinize research proposals, in particular those that do not seek to base themselves upon informed consent (i.e. utilising a public interest or a scientific research ground as described above). Ethics bodies may often be reticent to allow such research to proceed, in particular where there is not an extremely good reason for not using informed consent as a basis. This factor, taken together with the availability of the ‘legitimate interest’ ground for private entities (which is not available for public research institutions) and the comparative superior access that some commercial organisations may have to large pools of customer data mean that the de facto advantage public institutions possess over private sector competitors to conduct research is less than it would appear on a simple reading of the GDPR.

In other important contexts however this advantage may be more important given that the legitimate interests base it not sufficient alone to allow the processing of sensitive forms of data. This limitation also applies to the broad possibility for ‘further processing for scientific research’. This is an important limitation that should be taken into account when looking at the overall ability of private entities to conduct research with personal data. The use of sensitive data is indispensable to research in a number of areas that may have a high economic value and/or societal importance. Not being able to use the legal bases it can for research with non-sensitive data to conduct research with sensitive data means that private entities will often be forced to rely on explicit consent as a base for processing where universities may be able to use one of the research exceptions outlined in the GDPR. This may especially be the case in certain Member States where legislation has been formulated in a way so that the GDPR’s research exception is only available for research that is perceived to be in the ‘public interest’.

Given the continuing evolution of both big data and the research that it can facilitate this limitation of the legitimate interests base may become increasingly important, even in contexts where there is no express intention to use big data. This is because the larger a data set is, the greater the chances are that that the data in question will contain sensitive data. This is especially true with modern forms of big data that are at the heart many forms of innovative research. The never ending advances in computer power and associated refinement of analytical software will make it increasingly likely that sensitive data can be found within many forms of big data. Data that is seemingly innocuous and not personal in isolation may, when processed with other forms of data, reveal information that is personal and sensitive in nature. This problem will arguably become increasingly important for research in the future and mean that researchers (in both the public and private realms) will have to seek a legal base for processing sensitive data. This situation will arguably increase the importance of public/private sector collaboration in research in order to combine the data gathering abilities of the private sector with the research options foreseen within the GDPR for sensitive that are often more available for publicly funded research entities (where indeed such research in the public interest).

The author of this paper suggests that an urgent priority is to clarify the scope of make use of the seemingly extremely broad possibility the GDPR allows for ‘further processing for purposes of scientific research’. Further clarifications on this last ground are needed quickly given the concerningly apparent wide remit it presents data controllers to further process personal data for purposes of scientific research if they are already legally in possession of the data in question. It is unfortunate that such guidance was not provided in the EDPS opinion on scientific research. One can only hope that such guidance is forthcoming quickly.

## Data Availability

N/A

## Abbreviations

DPIA: Data protection impact assessment
DPO: Data Protection Officer
EDPS: European Data Protection Supervisor
HER: Electronic Health Record
EU: European Union
GDPR: General Data Protection Regulation

## Footnotes

1 For a rich an interesting analysis of the changing landscape in this area see: Corrales. M, Fenwick. M, Forgo. F, “New Technology, Big Data and the Law”, Springer, (2017). See also: Quinn. P, “Is the GDPR and Its Right to Data Portability a Major Enabler of Citizen Science?”, Global Jurist, (2018), DOI: 10.1515/gj-2018-0021

2 This is noticeable in fields such as genetics where study that is primarily computational in nature is becoming of increasing importance. See: Quinn. P & Quinn. L, “Big Genetic data and its big data protection challenges”, Computer Law and Security Review, (2018), 5, 34, p1000–1018

3 GDPR recital 26

4 As the author pointed out in a previous paper however, discerning whether data is truly anonymous may be a difficult task. In addition, where true anonymity is achieved it may often render data useless in research terms. See: Quinn. P, “The Anonymisation of Research Data — A Pyric Victory for Privacy that Should Not Be Pushed Too Hard by the EU Data Protection Framework?”, European Journal of Health Law, (2017), 24, 4, p1–21

5 The GDPR (in recital 26) unlike the previous directive 95/46/EC confirms explicitly that pseudonymized data (unlike anonymized data) is indeed personal data.

6 Article 29 Working Party Opinion 05/2014 on Anonymisation Techniques, Adopted on 10 April 2014, 0829/14/EN WP216

7 Directive 95/46/EC on the protection of individuals with regard to the processing of personal data and on the free movement of such data, is a European Union directive adopted in 1995 which regulates the processing of personal data within the European Union (EU). It was superseded by the GDPR, see Fn 23

8 Regulation (EU) 2016/679 of the European Parliament and of the Council of 27 April 2016 on the protection of natural persons with regard to the processing of personal data and on the free movement of such data, and repealing Directive 95/46/EC

9 Important exceptions remain, as will be discussed in section 5. These notably include exceptions for some forms of sensitive data such as health for which the GDPR (article 9(4)) allows an Member States to create further potentially divergent regulation.

10 Quinn. P & Quinn. L, “Big Genetic data and its big data protection challenges”, Computer Law and Security Review, (2018), 5, 34, p1000–1018 Despite the harmonizing effect of the GDPR, concerns still remain concerning the manner in which individual Member States are able to employ their own interpretation of key concepts. For more see: Peloquin. D, DiMaio. M, Bierer. B, Barnes, M, “Disruptive and avoidable: GDPR challenges to secondary research uses of data”, European Journal of Human Gentics, (2020), 28, 697–705

11 There are some important exceptions to the application of the GDPR however. This includes most criminal justice related activities. Article 2(2) for instances states that the regulation does not apply to activities “by competent authorities for the purposes of the prevention, investigation, detection or prosecution of criminal offences or the execution of criminal penalties, including the safeguarding against and the prevention of threats to public security.”

12 GDPR Recital 33

13 Data Subject rights are outlined in chapter 3 (articles 12–20) of the GDPR

14 Article 9(4) of the GDPR for example allows Member States to create additional requirements for the processing of various forms of sensitive data.

15 This is confirmed by Article 5(a) which calls for all processing of personal data to be “lawful”

16 Article 6 outlines consent as a legal base for the processing of data. Article 7 outlines some of the requirements attached to consent. As section 5 will discuss, article 9 outlines “explicit consent” as as legal basis for the processing of special categories of data.

17 Numerous concerns were raised on the part of research scientists during the lengthy negotiation process between various EU institutions for the GDPR. See for example: Nyren. O, Stenbeck. M, Groberg. H, “The European Parliament proposal for the new EU General Data Protection Regulation may severely restrict European epidemiological research”, European Journal of Epidemiology (2014), 29, p-227-230

18 GDPR Article 4(11)

19 “Under the GDPR, a data subject must inter alia be informed about the identity and contact details of the data controller, the data protection officer, the purposes for which the data will be processed, the recipients of the data, the duration of storage and the right to withdraw consent if consent is the lawful basis of processing”. See Staunton. C, Slokenberga. S, Mascalzoni. D, “The GDPR and the research exemption: considerations on the necessary safeguards for research biobanks”, European Journal of Genetics (2019), 27, p1159–1167

20 Article 29 Working Party Guidelines on consent under Regulation 2016/679 (2017) 17/EN WP259 p28: When regarded as a whole, the GDPR cannot be interpreted to allow for a controller to navigate around the key principle of specifying purposes for which consent of the data subject is asked

21 Corrigan. O, “Empty ethics: the problem with informed consent” Sociology of Health & Illness, 25, 3, p768–792; Solove. D, “Introduction: Privacy Self-Management and the Consent Dilemma” Harvard Law Review, (2013), 126, p1880–1903. GDPR recital 43 states “… In order to ensure that consent is freely given, consent should not provide a valid legal ground for the processing of personal data in a specific case where there is a clear imbalance between the data subject and the controller, in particular where the controller is a public authority and it is therefore unlikely that consent was freely given in all the circumstances of that specific situation”

22 The use of this legal base for research has been confirmed by the Article 29 working party (albeit discussing the applicability of Directive 95/46/EC). See: Article 29 Data Protection Working Party, Opinion 06/2014 on the notion of legitimate interests of the data controller under Article 7 of Directive 95/46/EC, European Commission, 9 April 2014 22. This matter is also discussed in Donnelly. M & McDonagh. M, “Health Research, Consent and the GDPR Exemption”, European Journal of Health Law, (2019), 26,2, p97–119 in section 3.1

23 GDPR Article 6(3)

24 The need for necessity and proportionality in the processing of personal data in the public interest has long been established by the European Court of Human Rights. A foundational case *was S and Marper v United Kingdom* [2008] ECHR 1581 where the requirements of necessity and proportionality were placed alongside the need for legality i.e. the existence of legislation outlining the processing in question.

25 One important area where this legal base can not be used is in research with sensitive (or special data). As section § discusses further, article 9, which outlines the bases for the processing of sensitive data does not include processing for contractual reasons. This means another base must be sought e.g. explicit consent. This could for example be the case with online commercial genealogical research databases. In such instances companies operating such a business model will need to also secure explicit consent to process sensitive data. (in addition to any contractual agreement they have to conduct research.)

26 For a broad (though pre GDPR) discussion of the concept of legitimate interests see: Article 29 Data Protection Working Party, Opinion 06/2014 on the notion of legitimate interests of the data controller under Article 7 of Directive 95/46/EC, European Commission, 9 April 2014

27 Donnelly. M & McDonagh. M, “Health Research, Consent and the GDPR Exemption”, European Journal of Health Law, (2019), 26,2, 97–119. The Article 29 working party did outline some potential room for public entities to use legitimate interests as a legal base where they are not carrying out a public function. (See WP29 Opinion on Legitimate Interests, p26). The author would argue that the applicability of this to research matters is likely to be limited.

28 See Article 29 Opinion on legitimate intersts, p.40

29 See Article 29 Opinion on legitimate interests, p.30

30 GDPR Article 14(5)b

31 Ducato. R, “Data protection, scientific research, and the role of information” CRIDES Working Paper Series, (2020) no. 1/2020, Computer Law and Security Review, forthcoming. Available at: file:///C:/Users/Pc/AppData/Local/Packages/Microsoft.MicrosoftEdge_8wekyb3d8bbwe/TempState/Downloads/2020%20-%20CRIDES%20WPS_1_2020_Ducato%20(1).pdf In this quote the author is referring to EDPS, Preliminary opinion on data protection and scientific research, p. 20.

32 Article 89(2) states “Where personal data are processed for scientific or historical research purposes or statistical purposes, Union or Member State law may provide for derogations from the rights referred to in Articles 15, 16, 18 and 21 subject to the conditions and safeguards referred to in paragraph 1 of this Article in so far as such rights are likely to render impossible or seriously impair the achievement of the specific purposes, and such derogations are necessary for the fulfilment of those purposes.

33 These rights are outlined in GDPR article 15, 16, 18 and 21 respectively.

34 The author discusses these difficulties in the context of genetic research in: Quinn. P & Quinn. L, “Big Genetic data and its big data protection challenges”, Computer Law and Security Review, (2018), 5, 34, p1000–1018

35 GDPR Article 89(2)

36 One important effect however of the requirement of national law, is that the extent to which rights are restricted may vary from one Member State to another, negating to a certain extent the general harmonizing mission of the GDPR. This means that the ability to limit data subject rights may vary considerably from one Member State to another.

37 See section 4.B where data subject rights are discussed

38 EDPS opinion on Scientific Research, page 16

39 The changing nature of sensitive data and its importance to researchers is reviewed by the author in and a colleague in Quinn. P & Malgeri. G, “Sensitive Data – Fast becoming a paper tiger (forthcoming)

40 This notion is extremely wide. See Article 29 Data Protection Working Party, Advice Paper on Special Categories Of Data (‘Sensitive Data’). See also annex to letter written by the Article 29 Working Party to the European Commissionon Feburary 5th 2014 concerning the interpretation of health data, available at: http://ec. europa.eu/justice/data-protection/article-29/documentation/ otherdocument/files/2015/20150205 _ letter _ art29wp _ ec _ health _ data _ after _ plenary _ annex _ en.pdf

41 For more on the complexities of performing data protection impact assessments in various circumstances see: Kloza, D, Van Dijk,N, Gellert, R, Böröcz, I, Tanas,A, Mantovani, E, Quinn, P. (Brussels Laboratory for Data Protection & Privacy Impact Assessments (d.pia.lab)),Data protection impact assessments in the European Union: complementing the new legal framework towards a more robust protection of individuals d.pia.lab PolicyBrief No.1/2017,2017,ISSN2565–9936.

42 For a discussion on the impact of such research in the are of genomics see Quinn. P & Quinn. L, “Big Genetic data and its big data protection challenges”, Computer Law and Security Review, (2018), 5, 34, p1000–1018

43 In Directive 95/46/EC the legal bases for the processing of sensitive data were outlined in article 8. For more see also: Quinn. P, Habbig. A, Mantovani. E, De Hert. P “The data protection and medical device frameworks—obstacles to the deployment of mHealth across Europe?“, European journal of health law, (2013) 20 (2), 185–204

44 GDPR Article 4(11) defines consent as Consent of the data subject means any freely given, specific, informed and unambiguous indication of the data subject’s wishes by which he or she, by a statement or by a clear affirmative action, signifies agreement to the processing of personal data relating to him or her.

45 GDPR Article 7(1)

46 For more on the consent/ anonymize dichotomy and the problems it can cause for research see: Carter., P Laurie. G, Dixon-Woods. M “The social licence for research: why care.data ran into trouble”, Journal of Medical Ethics, (2015), 41, p404–409

47 Hallinan. D & Friedewald. M,. “Open consent, biobanking and data protection law: can open consent be ‘informed’ under the forthcoming data protection regulation?”, Life Sciences, Society and Policy, (2015) 11:1. doi: 10.1186/s40504-014-0020-9; Quinn. P, “The Anonymisation of Research Data — A Pyric Victory for Privacy that Should Not Be Pushed Too Hard by the EU Data Protection Framework?”, European Journal of Health Law, (2017), 24,4, pp1–21

48 GDPR recital 26

49 The GDPR foresees legal bases for the processing of sensitive data for reasons of ‘substantial public interest’ (article 9(g) and ‘public health (article 9(i)).

50 For more see ‘EPDPS Opinion on Scientific Research’, p23

51 For more discussion of the concept of substantial public interest see: Hallinan. D, “Broad consent under the GDPR: an optimistic perspective on a bright future”, Life Sciences, Society and Policy, (2020), 16, 1, 10.1186/s40504-019-0096-3

52 EDPS opinion on scientific research, P19

53 For more on the potentials problems of publicly available research information in the context of genetic research see: Quinn. P & Quinn. L, “Big Genetic data and its big data protection challenges”, Computer Law and Security Review, (2018), 5, 34, p1000–1018

54 The EDPS for example notes cautiously “Publishing personal data in a biography or an article in the press is not the same as posting a message on a social media page.” See EDPS opinion on scientific research, p19

55 For a discussion on the German perspective for example see Molnár-Gábor. F, “Germany: a fair balance between scientific freedom and data subjects’ rights?” Human Genetics (2018) 137:619–626

56 The European Data Protection Supervisor recognised that some form of consent might even be desirable even where it is not the legal base relied upon for the processing of personal data. In such instances the use of consent is supplemental and mainly for ethical purposes. See EDPS opinion on scientific research, p20

57 For more see the discussion in Fn39 concerning recital 43 of the GDPR.

58 See sections 4 & 5.

59 In the UK for example, national legislation is seen as providing a broad discretion for public sector organizations to process genetic data without consent.

60 For an alternative view see the report composed by (EPRS) - European Parliamentary Research Service Scientific Foresight Unit (STOA) “How the General Data Protection Regulation changes the rules for scientific research” PE 634.447 – July 2019 p66

61 In the UK the Relevant Legislation is the Data Protection Act (2018). The UK’s information commissioner has also stated that researchers who want to use personal data without consent must establish that the research in question is in the public interest. See ICO report “Guide to the General Data Protection Regulation (GDPR)” (n 14), p 284. Available at: https://ico.org.uk/media/for-organisations/guide-to-the-general-data-protection-regulation-gdpr-1-0.pdf

62 In Ireland the primary legislation connected to the GDPR is the Data Protection Act 2018. It is accompanied by the Health Research Regulations 2018 which regulate research using health data. For further analysis see: Dove. E & Chen. J “Should consent for data processing be privileged in health research? A comparative legal analysis” International Data Privacy Law, (2019), 10.1093/idpl/ipz023 see p8-10 Under Irish law data controllers must seek permission from the minister of Health who will issue a decision *vis-à-vis* an appointed committee. An application must be accompanied by “written information demonstrating that the public interest in carrying out the health research significantly outweighs the public interest in requiring the explicit consent of the data subject under Regulation 3(1)(e) together with a statement setting out the reasons why it is not proposed to seek the consent of the data subject for the purposes of the health research.”

63 GDPR Articles 6(b) and 6(c)

64 GDPR Article 6(1)(f)

65 See: Article 29 Data Protection Working Party, Opinion 06/2014 on the notion of legitimate interests of the data controller under Article 7 of Directive 95/46/EC, European Commission, 9 April 2014, p40

66 This is issue was highlighted by the New York Times as far Back as 2012. See Markoff. J “Troves of Personal Data, Forbidden to Researchers”, Published 21 May 2012 Available at: https://www.nytimes.com/2012/05/22/science/big-data-troves-stay-forbidden-to-social-scientists.html

67 This has been demonstrated during the COVID-19 pandemic. See Hao. K, “How Facebook and Google are helping the CDC forecast coronavirus” Published Online on MIT Technology Review, 09 April 2020. Available at: https://www.technologyreview.com/2020/04/09/998924/facebook-and-google-share-data-to-forecast-coronavirus/

68 The same is true for other bases that, as section 4 discusses, could be potentially applicable to research also (e.g. processing to meet a contractual obligation).

69 See WP29 Opinion on Legitimate Interests, p15. The Working party stated (in the context of Directive 95/46/EC) “a controller processing special categories of data may never invoke solely a legal ground under Article 7 to legitimize a data processing activity. Where applicable, Article 7 will not prevail but always apply in a cumulative way with Article 8 to ensure that all relevant safeguards and measures are complied with”
